# Supplementary material for: The Evolution and Characterization of the RNA Interference Pathways in Lophotrochozoa
Source: Genome Biol Evol. 2024 May 7;16(5):evae098. doi: 10.1093/gbe/evae098 (PMC11114477; doi:10.1093/gbe/evae098)
Supplement: evae098_Supplementary_Data [file evae098_supplementary_data.zip › Supplementary_materials.zip!/selected_miRNAs/NoGe_selected_miRNAs/mirdeep_pdf_files/NMRB01000014.1_Notospermus_geniculatus_strain_Ushimado_scaffold14,_whole_genome_shotgun_sequence_631.pdf]

| | | |
|---|---|---|
| Provisional ID | : | NMRB01000014.1_Notospermus_geniculatus_strain_Ushimado_scaffold14,_whole_genome_shotgun_sequence_631 |
| Score total | : | 895.1 |
| Score for star read(s) | : | 3.9 |
| Score for read counts | : | 889.8 |
| Score for mfe | : | 2 |
| Score for randfold | : | 0 |
| Score for cons. seed | : | -0.6 |
| Total read count | : | 1757 |
| Mature read count | : | 1570 |
| Loop read count | : | 0 |
| Star read count | : | 187 |

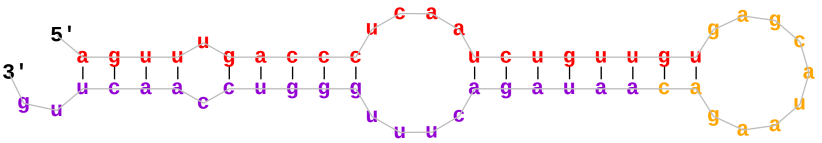


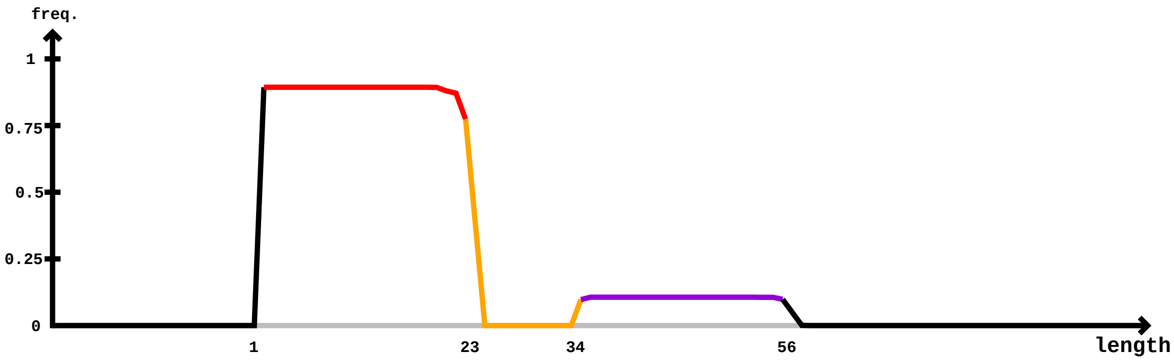


**Mature** **Star**

```
5'- uaccaauuccugagacggcgaguuugacccucaaucuguugugagcauaagacaauagacuuuggguccaacuugcuguuugaggagcaucaaacgggauacccaaaggucа -3'  obs
    uaccaauuccugagacggcgaguuugacccucaaucuguugugagcauaagacaauagacuuuggguccaacuugcuguuugaggagcaucaaacgggauacccaaaggucа       exp
    .(((..(((((.((((((((((((.(((((....((((((((.........))))))))....))))).)))))))))))).)))))........(((...)))...))).. reads  mm  sample
    ....................aguuugacccucaaucug..........................................................................  1  0  Mm3
    ....................Nguuugacccucaaucugu.........................................................................  1  1  Mm3
    ....................aguuugacccucaaucugu.........................................................................  1  0  Mm3
    ....................aguuugacccucaaucuguu........................................................................  1  0  Mm3
    ....................aguuugacccucaaucuguug.......................................................................  23  0  Mm3
    ....................aguuuAacccucaaucuguugu......................................................................  1  1  Mm3
    ....................aguuugacccucaaucuguugu......................................................................  116  0  Mm3
    ....................aguuugacccucaaucuCuugu......................................................................  1  1  Mm3
    ....................aguuugacccucaaucuguuguU.....................................................................  3  1  Mm3
    ....................aguuugacccucaaucuguuguA.....................................................................  2  1  Mm3
    .....................................................aauagacuuuggguccaac........................................  1  0  Mm3
    .....................................................aauagacuuuggguccaacuu......................................  2  0  Mm3
    .....................................................aauagacuuuggguccaacuug.....................................  11  0  Mm3
    .....................................................aauagacuuuggguccaacuugUu...................................  1  1  Mm3
    ......................................................auagacuuuggguccaacuugU....................................  1  1  Mm3

    ....................aguuugacccucaaucugu.........................................................................  9  0  Fm3
    ....................aguuugacccucaaucuguug.......................................................................  40  0  Fm3
    ....................aguuugacccCcaaucuguug.......................................................................  1  1  Fm3
    ....................aguuugacccucaaucuguuUu......................................................................  1  1  Fm3
    ....................aguuugacccucaaucuguugu......................................................................  339  0  Fm3
    ....................aguuugacccucaGucuguugu......................................................................  2  1  Fm3
    ....................aguuugacccucaaucAguugu......................................................................  1  1  Fm3
    ....................aguuugacccucaaucuCuugu......................................................................  1  1  Fm3
    ....................aguuugacccucaaucCguugu......................................................................  1  1  Fm3
    ....................aguuugacccucaaucuAuugu......................................................................  4  1  Fm3
    ....................aguuugacUcucaaucuguugu......................................................................  1  1  Fm3
    ....................aguuugacccucaaucuguuguU.....................................................................  11  1  Fm3
    .....................................................aauagacuuuggguccaacuu......................................  3  0  Fm3
    .....................................................aauagacuuuggguccaCcuug.....................................  1  1  Fm3
    .....................................................aauagacuuuggguccaacuug.....................................  8  0  Fm3
    .....................................................aauagacuuuggguccaacuugU....................................  3  1  Fm3
    ......................................................auagacuuuggguccaacuug.....................................  2  0  Fm3
```

**Mature** **Star**

uaccaauuccugagacggcgaguuugacccucaaucuguugugagcauaagacaauagacuuuggguccaacuugcuguuugaggagcaucaaacgggauacccaaagguca

| | | | |
|---|---|---|---|
| ....................aguuugacccucaaucugu......................................................................... | 2 | 0 | Mm2 |
| ....................aguuugacccucaaucuguug....................................................................... | 40 | 0 | Mm2 |
| ....................aguuugacccGcaaucuguugu...................................................................... | 1 | 1 | Mm2 |
| ....................aCuuugacccucaaucuguugu...................................................................... | 1 | 1 | Mm2 |
| ....................aguuugacccucaaucuguugu...................................................................... | 213 | 0 | Mm2 |
| ....................aguuugacccucaaucuCuugu...................................................................... | 1 | 1 | Mm2 |
| ....................aguuugacccucaaucuUuugu...................................................................... | 1 | 1 | Mm2 |
| ....................aguuugacccucaaucuAuugu...................................................................... | 2 | 1 | Mm2 |
| ....................aguuugacccucaaucCguugu...................................................................... | 1 | 1 | Mm2 |
| ....................aguuugacccucaGucuguugu...................................................................... | 1 | 1 | Mm2 |
| ....................aguuugacccucaaucuguuguU..................................................................... | 9 | 1 | Mm2 |
| ....................aguuugacccucaaucuguuguA..................................................................... | 4 | 1 | Mm2 |
| .....................................................aauagacuuuggguccaacuug..................................... | 22 | 0 | Mm2 |
| .....................................................aauagacuuuggguccaacuugU.................................... | 1 | 1 | Mm2 |
| .....................................................aauagacuuuggguccaacuugA.................................... | 1 | 1 | Mm2 |
| ......................................................auagacuuuggguccaacuu...................................... | 1 | 0 | Mm2 |
| ......................................................auagacuuuggguccaacuugU.................................... | 2 | 1 | Mm2 |
| ....................aguuugacccucaaucugu......................................................................... | 6 | 0 | Mm1 |
| ....................aguuugacccucaaucuguu........................................................................ | 7 | 0 | Mm1 |
| ....................aguuugaUccucaaucuguu........................................................................ | 1 | 1 | Mm1 |
| ....................aguuugacccucaaucuAuugu...................................................................... | 1 | 1 | Mm1 |
| ....................aguuugacccucaaucuguugu...................................................................... | 117 | 0 | Mm1 |
| ....................aguuugacccucaaucuCuugu...................................................................... | 2 | 1 | Mm1 |
| ....................aguuugacccucaaucAguugu...................................................................... | 1 | 1 | Mm1 |
| ....................aguuugacccAcaaucuguugu...................................................................... | 1 | 1 | Mm1 |
| ....................aguuugacccucaaucuguuguU..................................................................... | 1 | 1 | Mm1 |
| ....................aguuugacccucaaucuguuguA..................................................................... | 7 | 1 | Mm1 |
| .....................................................aauagacuuuggguccaacuug..................................... | 5 | 0 | Mm1 |
| .....................................................aauagacuuuggguccaacuugU.................................... | 2 | 1 | Mm1 |
| ....................aguuugacccGcaaucugu......................................................................... | 1 | 1 | Fm1 |
| ....................aguuugacccucaaucugu......................................................................... | 3 | 0 | Fm1 |
| ....................aguuugacccucaaucuguug....................................................................... | 55 | 0 | Fm1 |
| ....................aguuugaccAucaaucuguugu...................................................................... | 1 | 1 | Fm1 |
| ....................aguuugaUccucaaucuguugu...................................................................... | 6 | 1 | Fm1 |
| ....................Uguuugacccucaaucuguugu...................................................................... | 1 | 1 | Fm1 |
| ....................aguuugacccucaaucuguugG...................................................................... | 1 | 1 | Fm1 |
| ....................aguuugacccucaaucuUuugu...................................................................... | 1 | 1 | Fm1 |
| ....................aguuugacccucaaucuAuugu...................................................................... | 3 | 1 | Fm1 |
| ....................aguuugacccGcaaucuguugu...................................................................... | 1 | 1 | Fm1 |
| ....................aguuugacccAcaaucuguugu...................................................................... | 1 | 1 | Fm1 |
| ....................aguuugacccucaaucCguugu...................................................................... | 2 | 1 | Fm1 |
| ....................aguuugacccucaaucAguugu...................................................................... | 1 | 1 | Fm1 |
| ....................aguuugacccucaaucuCuugu...................................................................... | 1 | 1 | Fm1 |
| ....................aguuugacccucaaucuguugu...................................................................... | 293 | 0 | Fm1 |
| ....................aguuugacccucaaucuguuguU..................................................................... | 18 | 1 | Fm1 |
| ....................aguuugacccucaaucuguuguA..................................................................... | 4 | 1 | Fm1 |
| .....................................................aauagacuuuggguccCacuu...................................... | 1 | 1 | Fm1 |
| .....................................................aauagacuuuggguccaacuu...................................... | 6 | 0 | Fm1 |
| .....................................................aauagacuuuggguccaacuug..................................... | 69 | 0 | Fm1 |
| .....................................................aauagacuuuggguccaacuGg..................................... | 1 | 1 | Fm1 |
| .....................................................aauagacuuuggguccaacuuU..................................... | 1 | 1 | Fm1 |
| .....................................................Uauagacuuuggguccaacuug..................................... | 1 | 1 | Fm1 |
| .....................................................aauagacuuuggguccaacuugU.................................... | 16 | 1 | Fm1 |
| ......................................................auagacuuuggguccaacuug..................................... | 4 | 0 | Fm1 |
| ......................................................auagacuuuggguccaacuugU.................................... | 6 | 1 | Fm1 |
| ....................aguuugacccucaaucuguu........................................................................ | 6 | 0 | Fm2 |
| ....................aguuugacccucaaucuguug....................................................................... | 13 | 0 | Fm2 |
| ....................aguuugacccucaaucuAuugu...................................................................... | 2 | 1 | Fm2 |
| ....................aguuugacccucaaucuCuugu...................................................................... | 1 | 1 | Fm2 |
| ....................aguuugacccucaaucCguugu...................................................................... | 1 | 1 | Fm2 |
| ....................aguuugacccucaaucuguugu...................................................................... | 172 | 0 | Fm2 |
| ....................aguuugacccucaaucuguugG...................................................................... | 1 | 1 | Fm2 |
| .....................................................aauagacuuuggguccaacuug..................................... | 15 | 0 | Fm2 |
